# Aortoiliac arterial thrombosis and renal artery stenosis in a patient with neonatal multisystem inflammatory syndrome: a case report and review of literature

**DOI:** 10.3389/fped.2024.1474192

**Published:** 2025-01-10

**Authors:** Oranooj Lertkovit, Daranee Isaranimitkul, Suwanna Pornrattanarungsri, Ornatcha Sirimongkolchaiyakul, Sirikarn Tangcheewinsirikul, Pichada Saengrat

**Affiliations:** ^1^ Division of Hematology and Oncology, Department of Pediatrics, Faculty of Medicine Vajira Hospital, Navamindradhiraj University, Bangkok, Thailand; ^2^ Division of Cardiology, Department of Pediatrics, Faculty of Medicine Vajira Hospital, Navamindradhiraj University, Bangkok, Thailand; ^3^ Division of Nephrology, Department of Pediatrics, Faculty of Medicine Vajira Hospital, Navamindradhiraj University, Bangkok, Thailand; ^4^ Division of Rheumatology, Department of Pediatrics, Faculty of Medicine Vajira Hospital, Navamindradhiraj University, Bangkok, Thailand; ^5^ Division of Neonatology, Department of Pediatrics, Faculty of Medicine Vajira Hospital, Navamindradhiraj University, Bangkok, Thailand

**Keywords:** MIS-N, case report, neonatal hypertension, thrombosis, SARS-CoV-2

## Abstract

**Background:**

Multisystem inflammatory syndrome in neonates (MIS-N) is a rare condition thought to be associated with prenatal exposure to maternal severe acute respiratory syndrome coronavirus 2 infection. This immune-mediated hyperinflammation has been described in neonates with multiorgan dysfunction, including cardiopulmonary, encephalopathy, coagulopathy, and vascular complications. However, renovascular complications in MIS-N are rare. Here, we present a case with aortoiliac arterial thrombosis and renovascular stenosis in MIS-N.

**Case presentation:**

A 2-day-old, full-term, male neonate presented with fever, respiratory failure, hypotensive shock, and elevated inflammatory markers. He was transferred to our neonatal intensive care unit for comprehensive monitoring and treated with antibiotics for early-onset neonatal sepsis. However, his clinical condition deteriorated. His mother reported a history of severe acute respiratory syndrome coronavirus 2 infection during the late second trimester. He was diagnosed with MIS-N based on the fulfillment of the diagnostic criteria for multiorgan involvement and laboratory findings. He responded to intravenous immunoglobulin, corticosteroids, and aspirin. However, he later developed significant hypertension, which was associated with aortoiliac arterial thrombosis and right renal artery stenosis. An elevated ferritin level was observed. The ongoing inflammatory condition was considered, and corticosteroids were restarted along with treatment for documented thrombosis using enoxaparin and aspirin. After treatment, partial resolution of aortoiliac arterial thrombus was observed. However, narrowing of the proximal part of right renal artery persisted, and blood pressure remained difficult to control, requiring multiple antihypertensive agents. Finally, the patient underwent percutaneous transluminal balloon angioplasty to control hypertension.

**Conclusion:**

Our case revealed the clinical course of MIS-N with renovascular complications. The condition's hyperinflammatory state may have played a pathophysiological role in the development of this life-threatening complication. Although there is an increased risk of bleeding in MIS-N, thromboprophylaxis should be considered in high-risk patients. An early multidisciplinary approach is recommended to ensure prompt diagnosis and improve outcomes.

## 1 Introduction

Multisystem inflammatory syndrome in neonates (MIS-N) has been increasingly reported among neonates with prenatal exposure to maternal severe acute respiratory syndrome coronavirus 2 (SARS-CoV-2) infection or early postnatal exposure to SARS-CoV-2 infection (1). Transplacental exposure to SARS-CoV-2 antibodies and cytokines or fetal infection may lead to the development of hyperinflammatory response among neonates (2). A recent systematic review has suggested the diagnostic criteria for MIS-N, which are mainly based on the criteria for the diagnosis of multisystem inflammatory syndrome in children (MIS-C) (3). The common clinical manifestations of MIS-N include cardiac dysfunction, arrhythmia, dilated coronaries, respiratory distress, persistent pulmonary hypertension, encephalopathy, and coagulopathy (2, 4). However, the gastrointestinal manifestations and fever that are commonly observed in MIS-C are rarely reported in MIS-N. Previous studies reported a significant hypercoagulable state, vascular complications, and increased risk of thrombosis among children with multisystem inflammatory syndrome, particularly in the high-risk group (patients aged ≥12 years, those with cancer, those with D-dimer levels >5× the upper normal limit, and those with a central venous catheter) (5–7). Among recent studies in children with multisystem inflammatory syndrome and SARS-CoV-2 infection, renovascular involvement has been infrequently reported (8).

Neonatal hypertension is rare with an incidence in the range of 0.8%–2% (9). Renovascular disease, including thromboembolism associated with umbilical arterial catheter placement and renal artery stenosis, is one of the common causes of neonatal hypertension (9, 10). The present case report describes MIS-N in a full-term newborn complicated with hypertension associated with aortoiliac arterial thrombosis and renal artery stenosis. Our report presents the clinical course, diagnosis timelines, and consequent outcomes of this disease. Moreover, the challenges in managing renovascular hypertension in a newborn diagnosed with MIS-N and the knowledge gaps of thromboprophylaxis that need to be explored are discussed.

## 2 Case description

A 2-day-old, full-term, male neonate was referred to our tertiary care unit with pneumothorax, respiratory failure, and shock. He was delivered via cesarean section because of maternal pre-eclampsia with severe features at 38 weeks of gestation. His Apgar scores were 8, 9, and 10 at 1, 5, and 10 min, respectively, and his birth body weight was 2,886 g. During the first hour of life, he experienced respiratory distress and developed progressive respiratory failure and hypotension. A chest radiograph revealed a right pneumothorax. The patient was then referred to our neonatal intensive care unit (NICU) after initial management, including endotracheal intubation, right intercostal drainage placement, umbilical vein catheterization, umbilical arterial catheter, and empirical antibiotics.

At the neonatal intensive care unit, he was critically ill with fever, respiratory failure, and hypotensive shock. Initially, neonatal sepsis investigations showed unremarkable hemoglobin (16.7 g/dl) and platelet levels (183,000/L), but his C-reactive protein (CRP) (22.3 mg/L), procalcitonin (34.7 μg/L), and white blood cell levels (total white blood cell count 21,670/mm^3^) were increased. Chest radiography revealed improved pneumothorax, proper position of the umbilical arterial catheter (high thoracic position), and umbilical vein catheterization. A cardiologist was consulted on day of life (DOL) 2 and performed echocardiography, which revealed pulmonary hypertension with normal coronary artery. The patient was managed with inhaled nitric oxide, high-frequency oscillatory ventilation support, hydrocortisone, broad-spectrum antibiotics, and multiple inotropic drugs (including adrenaline, noradrenaline, dobutamine, dopamine, and milrinone). MIS-N was considered as the patient had multiorgan involvement and partially responded to intensive therapy, together with a history of symptomatic SARS-CoV-2 infection with a positive SARS-CoV-2 antigen testing during the late second trimester of pregnancy in his mother. The patient tested positive for SARS-CoV-2 immunoglobulin G (IgG) (anti-nucleocapsid IgG level: 2.21) and negative for SARS-CoV-2 spike immunoglobulin M and antigen test.

**Figure 1:**
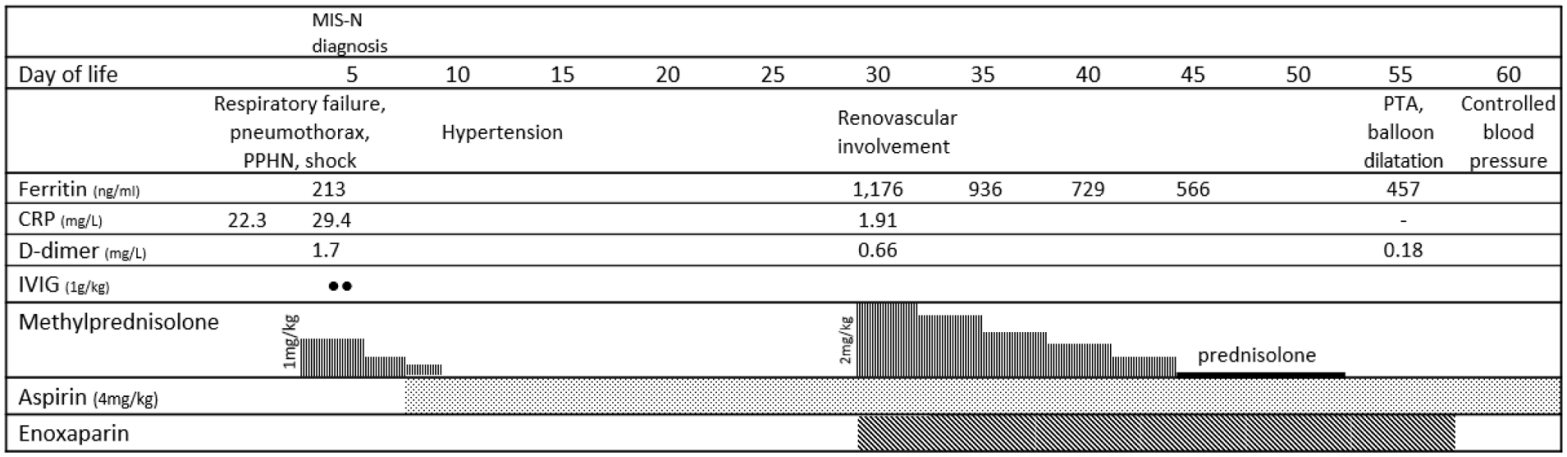
Clinical manifestation, essential laboratory findings, and key management of the patient. MIS-N, multisystem inflammatory syndrome in neonates; PPHN, persistent pulmonary hypertension of the newborn; PTA, percutaneous transluminal angioplasty; CRP, C-reactive protein; IVIG, intravenous immunoglobulin.

Laboratory evaluation revealed elevated cardiac enzymes (troponin-T, 410 ng/L; pro B-type natriuretic peptide, >35,000 pg/ml) and inflammatory markers (CRP, procalcitonin, lactate dehydrogenase, and interleukin-6). However, no microbial pathogens were identified during the evaluation. Despite broadening the antimicrobial regimen to include vancomycin and meropenem, aggressive management with multiple blood components, and higher vasopressors doses to maintain adequate perfusion, the patient deteriorated. A presumptive diagnosis of MIS-N was made after the patient met the clinical and laboratory criteria (Table 1) (2). MIS-N treatment was initiated with intravenous immunoglobulin (IVIG) and high-dose methylprednisolone, as shown in Figure 1.

**Table 1:** Diagnostic and patient-specific criteria of MIS-N.

Criteria	Our patient	
A neonate aged <28 days at the time of presentation	Yes	Early presentation within 24 h after birth
Laboratory or epidemiologic evidence of SARS-CoV-2 infection in the mother	Yes	Antenatal history of maternal upper respiratory tract infection with positive SARS-CoV-2 antigen test during the second trimester
	Yes	Serological evidence (positive SARS-CoV-2 anti-nucleocapsid IgG but not IgM) in the neonate
Clinical criteria		
– Severe illness necessitating hospitalization – Multisystem involvement – OR Cardiac AV conduction abnormalities OR coronary dilation or aneurysms (without involvement of a second organ system)	Yes Yes Yes	Cardiovascular, pulmonary, and coronary involvement
Laboratory evidence of inflammation		
– One or more of the following: an elevated CRP, ESR, fibrinogen, procalcitonin, D-dimer, ferritin, LDH, or IL-6; elevated neutrophils or reduced lymphocytes; low albumin	Yes	Elevated CRP (22.3 mg/L), procalcitonin (34.7 μg/L), LDH (1,204 U/L), IL-6 (152 pg/ml), and ferritin (1,176 ng/ml) Low albumin (2.7 g/ml)
No alternative diagnosis	Yes	

MIS-N, multisystem inflammatory syndrome in neonates; SARS-CoV-2, severe acute respiratory syndrome coronavirus 2; AV, atrioventricular; CRP, C-reactive protein; ESR, erythrocyte sedimentation rate; LDH, lactate dehydrogenase; IL-6, interleukin-6.

The follow-up echocardiogram revealed a new left coronary artery aneurysm [left main coronary artery (LMCA) Z-score 3.7; left anterior descending artery (LAD) Z-score 3.79], and normal ventricular function (ejection fraction 55%). The findings were consistent with coronary involvement in MIS-N. Hence, the patient was given aspirin. Thromboprophylaxis was not initiated because of concern for clinically gastrointestinal hemorrhage. After receiving IVIG and methylprednisolone, the patient improved rapidly, with fever resolution, progressively reduced respiratory support needs, and successful weaning off inotropes in 48 h.

**Figure 2:**
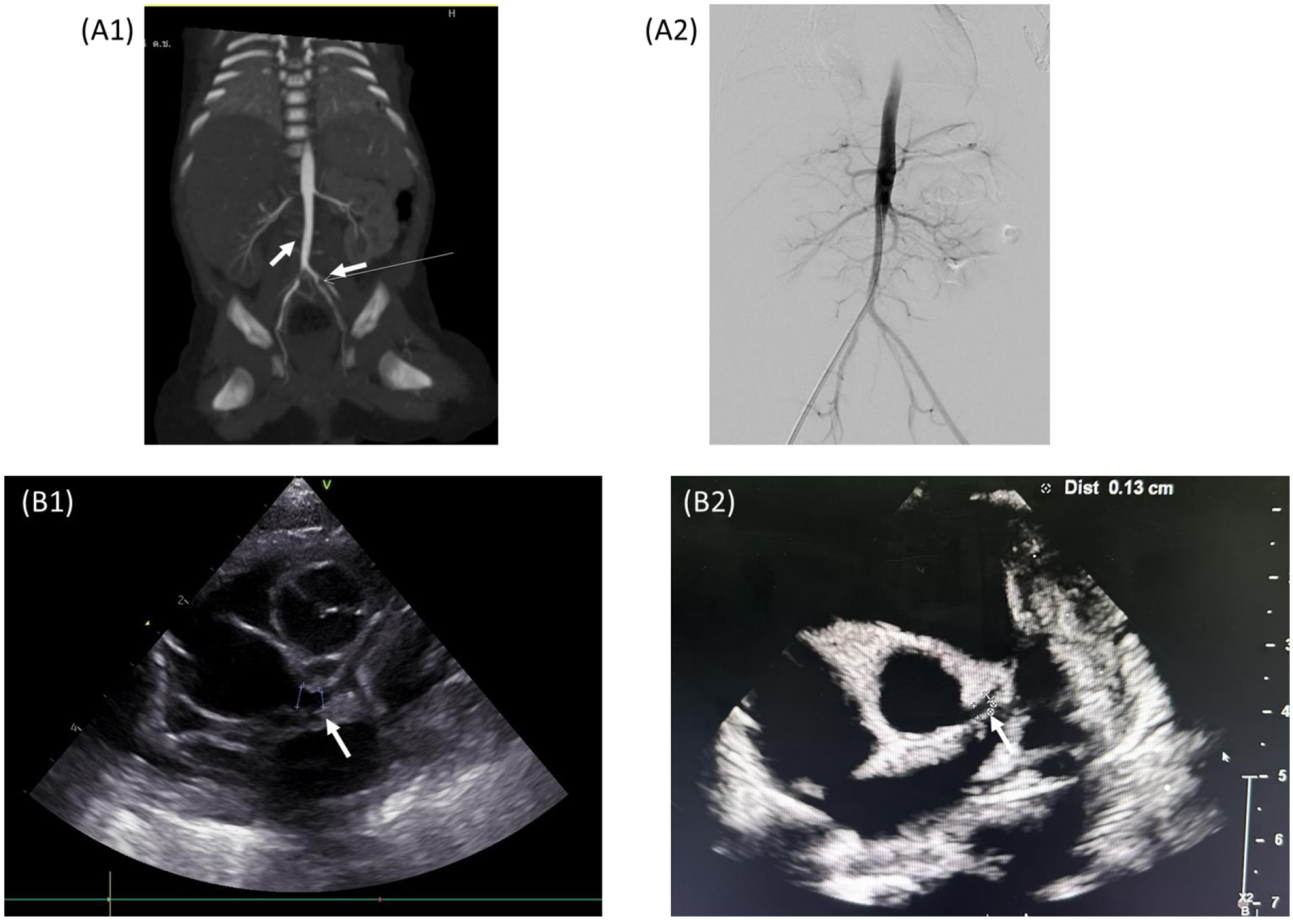
**(A1)** CTA abdominal aorta showing right renal artery stenosis and arterial thrombus in the juxta-renal abdominal aorta and left IIA. **(A2)** Angiography showing thrombus resolution at 3-week follow-up. **(B1)** Echocardiography at DOL 8 showing a new left coronary artery aneurysm. **(B2)** Echocardiography at age of 7 months showing regression of the coronary artery aneurysm to normal.

On DOL 11, the patient developed persistent hypertension. His systolic blood pressure rose to 130 mmHg (>95th percentile) despite the gradual tapering off of methylprednisolone. Initial laboratory tests to evaluate the cause of hypertension, including creatine, electrolytes, and urinalysis, showed unremarkable results. Renal Doppler ultrasonography was performed on DOL 21, which revealed a suspected filling defect of the infrarenal abdominal aorta. To confirm the presence of the suspected renovascular disease, computed tomography angiography (CTA) was performed, which showed a narrowing of the proximal right renal artery and a filling defect in the juxta-renal abdominal aorta and left internal iliac artery suggestive of thrombosis with delayed flow in the left internal iliac artery (Figure 2). The laboratory test showed a significant rise in ferritin levels, from 213 to 1,176 ng/ml, while CRP, LDH, and IL-6 levels returned to normal. Coagulation tests showed a drop in D-dimer levels from 1.7 mg/L at MIS-N diagnosis to 0.66 mg/L, with normal prothrombin time (PT) (11.7 s) and activated partial thromboplastin time (aPTT) (32.2 s). Protein C, protein S, and antithrombin III results were normal (77%, 79%, and 103%, respectively). His mother and other family members did not have any medical history of thrombotic disease. Subsequent echocardiography showed a persistent left coronary artery aneurysm (LMCA Z-score 3.94; LAD Z-score 2.73). The ongoing inflammatory process was considered and a high dose of methylprednisolone was restarted. The hematology team was consulted and enoxaparin was initiated (Figure 1). The dose was adjusted by monitoring anti-Xa activity (keeping the therapeutic level of 0.5–1.0 U/ml). The neonatal hypertension was partially controlled by multiple antihypertensive agents, including propranolol, hydralazine, and prazosin.

On DOL 41, follow-up magnetic resonance angiography of the whole aorta suggested partial resolution of infrarenal abdominal aorta thrombus, and complete resolution of left internal iliac artery thrombus. Still, narrowing of the proximal part of right renal artery persisted. Methylprednisolone was switched to prednisolone, while enoxaparin and aspirin were continued. The patient’s blood pressure remained poorly controlled, necessitating multiple antihypertensive medications. Finally, the patient underwent percutaneous transluminal balloon angioplasty dilatation for the right renal artery. The procedure findings revealed minimal renal artery stenosis and complete resolution of intraluminal thrombus. After the procedure, hypertension was controlled, permitting the reduction in the use of antihypertensive agents. The neonate received anticoagulation for a total of 4 weeks, methylprednisolone, with a gradually tapered dose, and discontinued after 4 weeks. He was discharged on DOL 61 with aspirin and propranolol. At the 1-month follow-up, he was doing well with adequate weight gain and normal blood pressure. Serial echocardiograms were performed after treatment at 1 week, 8 weeks, and then every 2 months. At 7 months, the echocardiogram revealed complete regression of the coronary artery aneurysm and aspirin was stopped.

## 3 Discussion

This case report presents a full-term, male newborn who presented with fever, respiratory failure, persistent pulmonary hypertension, hypotensive shock, elevated inflammatory markers, and a maternal history of SARS-CoV-2 infection 3 months before delivery, which met the proposed criteria for diagnosing MIS-N (2, 3). Commonly, MIS-N occurs in neonates after maternal SARS-CoV-2 infection 1–5 weeks before delivery. Some neonates can also develop MIS-N along with the maternal history of SARS-CoV-2 infection during the first or second trimester (11). The pathogenicity of MIS-N is not clearly understood, but proposed mechanisms include fetal exposure to transplacental maternal IgG antibodies or *in utero* infection of SARS-CoV-2, leading to immune dysregulation and multisystem inflammation in neonates (2, 4). Many of the laboratory criteria, including increased inflammatory markers, such as CRP, procalcitonin, and ferritin, are not specific to MIS-N and can also occur in severe sepsis. Our patients underwent multiple cultures, all showing no growth. Abnormal coronary artery appears to be the most specific clinical finding for the diagnosis of MIS-N (2, 12). The patient's clinical manifestations included cardiopulmonary and coronary artery involvement, complicated by significant hypertension. This eventually led to the discovery of aortoiliac arterial thrombosis and renal artery stenosis. His significant risk factors for thrombosis included the use of umbilical catheters, mechanical ventilation, and MIS-N.

Patients with MIS-N experience an immune-mediated hyperinflammatory state (3). This condition can lead to coagulation abnormalities, endothelial dysfunction, platelet activation, complement hyperactivation, and a potential predisposition to thromboembolism (13, 14). Previous studies have reported that despite the prothrombotic state, children with SARS-CoV-2 infection or multisystemic inflammatory syndrome rarely develop thrombotic complications (5, 6, 15), yet they have significantly increased mortality (12%). Currently, the incidence of thromboembolism events among patients with MIS-N remains unknown. Thromboembolism complications in MIS-N have also been described. A wide range of thromboembolism manifestations can occur, including arterial thrombosis, intracardiac thrombus, pulmonary thrombosis, and cerebral venous thrombosis, as previously reported in small case series and case reports (see Table 2). Similar to previous reports (12, 16, 18–20), arterial thrombosis was more frequently observed.

**Table 2:** Reported cases of MIS-N with diagnosed thrombosis complications.

Reference	Sex	Gestation (weeks)	Age at presentation	TE events	Treatment	Outcome	Comment
Pawar et al., India, 2021 (12)	M	36	Day 1	Large thrombus at left pulmonary artery origin on DOL 3	LMWH, alteplase, aspirin, inotropes, IVIG, steroids	Alive, complete resolution of clot at 8 weeks echo	
	F	38	Day 1	Intracardiac thrombus on DOL 4	LMWH for 6 weeks, IVIG, steroids	Alive, thrombus deceased in size at 4 weeks echo	
Amonkar et al., India, 2021 (16)	M	40	Day 6	Abdominal aorta thrombosis (80%–90% occlusion) with an ischemic right lower limb	Heparin, r-TPA, embolectomy, steroids, aspirin	Alive, below-knee amputation on DOL 16	Most likely MIS-N
Campi et al., Italy, 2021 (17)	–	39	Day 4	Right thalamic infarction secondary to thrombosis of the internal cerebral veins, thrombosis of the Galen vein and venous sinuses confluence, IVH	LMWH	Alive	Possible MIS-N
Gupta et al., India, 2022 (18)	F	Term	At birth	aortic and intracardiac thrombosis on DOL 6	IVIG, enoxaparin, aspirin	Alive	
Abdulaziz-Opiela et al., Poland, 2023 (19)	M	40	Day 1	Pulmonary emboli in the arterial duct, left pulmonary artery, and pulmonary trunk	LMWH	Alive, complete resolution of clot at 9th and 12th day of life echo	Possible MIS-N
Vijay et al., India, 2024 (20)	F	38	At birth	Right axillary and brachial arteries thrombosis	Heparin then enoxaparin	Alive, clinical improvement	Possible MIS-N
Our case, Thailand, 2024	M	38	Day 1	Aortoiliac arterial thrombosis and renal artery stenosis	IVIG, steroids, enoxaparin, aspirin	Alive	

MIS-N, multisystem inflammatory syndrome in neonate; TE, thromboembolism events; M, male; F, female; DOL, day of life; LMWH, low molecular weight heparin; IVIG, intravenous immunoglobulin; r-TPA, recombinant tissue plasminogen activator; IVH, intraventricular hemorrhage.

To the best of our knowledge, MIS-N causes vasculopathy and coronary dilatation (2). Moreover, there is a possibility that healing of the injured vascular wall may lead to vascular stenosis. Mishra et al. (21) published a report of two Indian infants with MIS-C and renal artery stenosis. The first case was a 13-month-old boy who presented with heart failure and hypertension. An echocardiogram showed severe left ventricular dysfunction with a normal coronary artery and CTA revealed left renal artery occlusion. The patient was successfully treated with IVIG, steroids, and balloon angioplasty dilatation. The second case was a 12-month-old girl who had a recent SARS-CoV-2 infection and then developed clinical heart failure and hypertension. Echocardiogram revealed dilated left ventricular with normal coronary artery and CTA confirmed the right renal artery occlusion with mural thickening. Unfortunately, it remains unclear whether the patient's renal artery stenosis was primary or secondary by MIS-C or a pre-existing underlying condition (e.g., infantile Takayasu arteritis).

The clinical guidelines of the American College of Rheumatology recommend evidence-based management for children with multisystemic inflammatory syndrome (22). Besides immunomodulatory treatment, including IVIG and glucocorticoid treatment, the American College of Rheumatology also recommends antiplatelet and anticoagulation therapy. Patients with MIS-C should receive low-dose aspirin, whereas those with poor ventricular function (ejection fraction <35%), documented thrombosis, and coronary artery aneurysm (maximal Z-score ≥10.0) should receive low-dose aspirin and therapeutic anticoagulation. However, there are no adequate studies for MIS-N. According to a recent systematic review (11), most neonates with MIS-N also responded well to immunomodulation therapy (intravenous glucocorticoid and/or IVIG) and required intensive care, including inotropic medications and respiratory support, for severe clinical manifestations. The use of anticoagulants and antiplatelet agents varied across studies, with anticoagulants primarily initiated in neonates experiencing thromboembolism events (4, 11).

In our case, there was cardiopulmonary and coronary artery involvement. The patient responded to initial immunomodulatory therapy with IVIG and methylprednisolone. Low-dose aspirin was prescribed for the treatment of coronary artery aneurysm. Later, the patient developed hypertension and CTA demonstrated proximal right renal artery stenosis and evidence of aortoiliac arterial thrombosis. Thrombophilia screening was normal. There was also an increasing trend of follow-up inflammatory markers. Steroids were restarted and documented thrombosis was treated with enoxaparin and aspirin. The patient responded to treatment, but a vascular procedure with percutaneous balloon angioplasty was required for the management of persistent renal stenosis.

This case highlights the unusual thrombosis and renovascular manifestations of neonates with prenatal exposure to SARS-CoV-2 infection. The case details are expected to provide necessary information for the diagnosis and management of MIS-N cases in the future.

## 4 Conclusions

We report the clinical course and timeline of renovascular hypertension as a result of COVID-19-associated MIS-N in a neonate. The cause of aortoiliac arterial thrombosis and renal artery stenosis in this patient was likely multifactorial, with potential risk factors, including umbilical catheterization, mechanical ventilation, and MIS-N. Further studies on the diagnosis, pathophysiology, management, disease monitoring, and sequelae of MIS-N are needed to provide further insights into this emerging condition. Moreover, thromboprophylaxis and bleeding risk assessment should be considered in patients with MIS-N.

## Data Availability

The raw data supporting the conclusions of this article will be made available by the authors, without undue reservation.
